# Label-Free Continuous Cell Sorting Using Optofluidic Chip

**DOI:** 10.3390/mi15070818

**Published:** 2024-06-25

**Authors:** Yingjie Zhang, Tao Zhang, Xinchun Zhang, Jingmeng Cheng, Sixiang Zhang

**Affiliations:** 1 Department of Mechanical Engineering, North China Electric Power University, Baoding 071003, China; 13095585725@163.com; 2 School of Mechanical Engineering, Hebei University of Technology, Tianjin 300000, China; cheng-jingmeng@hebut.edu.cn (J.C.); zhang-sixiang@hebut.edu.cn (S.Z.)

**Keywords:** T-matrix, cell sorting, optofluidics, high viability, scattering force, label-free, chip

## Abstract

In the field of biomedicine, efficiently and non-invasively isolating target cells has always been one of the core challenges. Optical fiber tweezers offer precise and non-invasive manipulation of cells within a medium and can be easily integrated with microfluidic systems. Therefore, this paper investigated the mechanism of cell manipulation using scattering force with optical fiber tweezers. We employed flat-ended single-mode fiber to drive and sort cells and derived the corresponding scattering force formula based on the T-matrix model. A single-mode optical tweezers system for cell sorting was developed, and an optofluidic experimental platform was constructed that effectively integrates the optical system with microfluidic chips. The chip, featuring an expanded cross-channel design, successfully achieved continuous separation of yeast cells (8~10 µm in diameter) and polystyrene microspheres (15~20 µm in diameter), with a sorting efficiency of up to 86% and maintaining viability in approximately 90% of the yeast cells. Compared to other sorting systems, this system does not require labeling and can achieve continuous sorting with cell viability at a lower cost of instrumentation.

## 1. Introduction

In the fields of biomedicine and chemical analysis, non-invasive and accurate sorting of microparticles and living cells is one of the focal points of multidisciplinary research [1]. Currently, microfluidic-based cell sorting technologies are experiencing rapid growth due to their exceptional attributes, which include superior efficiency, high throughput, high precision, low cost, integrability, and portability. Various microfluidic approaches for particle sorting primarily include dielectrophoretic [2,3,4,5,6], optical [7,8,9], acoustic [10,11,12,13], magnetic [14,15,16,17], and inertial techniques [18,19,20]. Among these, optical tweezers technology allows for the contactless manipulation of microparticles in a liquid environment using laser beams, making it particularly suitable for non-invasive operation and precise control of living cells. Optical tweezers also possess the capability to integrate with other biological and technical methods, enabling multifunctionality and real-time dynamic monitoring [21,22,23]. However, traditional optical tweezer technology also has some inevitable limitations, such as a complex optical system, limited number of manipulated particles, and low capture or sorting efficiency. In contrast, the use of optical fiber tweezers for cell manipulation avoids the limitations of fixed optical traps, offering greater freedom of manipulation and more observational dimensions. It also allows for direct access into the sample chamber, enabling cells to be manipulated freely within the medium. When combined with microfluidic technology, the miniaturization and integration of optical tweezer systems can be achieved. Therefore, the technology of manipulating particles with optical fiber tweezers is gradually becoming a trend in cell sorting.

To achieve stable manipulation of microparticles using optical fiber, researchers have utilized various fiber microfabrication techniques [24,25,26,27] to create fiber-optic tweezers and apply them to the capture and manipulation of microscopic particles [28,29,30]. Liu et al. [31] employed evanescent wave technology to securely capture nanoparticles and cells on the surface of an optical conveyor belt constructed from natural cellular materials. By adjusting the relative power of the laser and introducing counter-propagating dual beams, particles and cells can be conveyed in both directions along the biological belt, with adjustable transit lengths. Mumati et al. [32] proposed a method for capturing microparticles using photothermal swimming with a 2 µm wavelength thulium-doped fiber laser. However, this thermal effect could cause damage to biological cells and tissues. Zhao et al. [33] introduced an optical micromanipulator based on a four-core fiber that can form symmetrical dual optical tweezers about the fiber axis, enabling oscillatory manipulation of particles. Tang et al. [34] integrated three equivalent double-core fiber tweezers into a single seven-core fiber, processing the frustum into a hexagonal pyramid through fiber grinding. This seven-core fiber tweezers generates three discrete optical potential wells along the fiber’s main axis. Manipulating the incident laser power for each core allows for particle entrapment, long-distance traction, bidirectional movement, and control over axial placement. Deng et al. [35] fabricated an optical-fiber-based optical gun that processed a coaxial dual-waveguide fiber into a conical table-shaped fiber probe through fiber grinding technology, achieving three-dimensional capture of microparticles. Liu et al. [36] proposed and demonstrated dual-function optical tweezers operating on a single gradient-index multimode fiber. They created a special tapered fiber tip, and by adjusting the beam’s propagation mode within the gradient index multimode fiber, they achieved the first functional non-contact capture of particles and axial position adjustment among other operations. Zhang et al. [37] proposed all-fiber Bessel beam tweezers for three-dimensional capture of multiple particles, processing the fiber probe into a special semi-ellipsoidal structure. By splicing single-mode and step-index multimode fibers coaxially, they modulated the Bessel beam at the specially designed fiber probe tip, achieving three-dimensional capture of multiple particles. Tang et al. [38] spliced a section of single-mode fiber with a section of multimode fiber, and by assembling high refractive index spheres with large radius of curvature, they modulated the fiber Bessel beam, achieving non-contact, three-dimensional, and stable capture of individual nanoparticles. Zhang et al. [39] utilized the non-diffracting self-healing properties of Bessel beams to achieve multipoint capture and maneuver absorbent particles within a liquid medium.

Optofluidic technology combines the good transport characteristics of microfluidic systems for particles with optical tweezers technology, enabling many functions not available with static optical tweezers, one of which is particle screening. A team from the University of St Andrews in the UK [40] used two different wavelengths of lasers to generate counter-propagating evanescent fields, producing opposite optical forces on two different sizes of metal particles through plasmonic resonance, achieving particle separation. A team from Nanyang Technological University in Singapore [41] produced quasi-Bessel beams with microlenses integrated into optofluidic chips, constructing a “loose overdamped system” and successfully separating nanoparticles of different sizes. Subsequently, this team [42], based on Kramer’s single-particle hopping theory, revealed the mechanism of multi-particle hopping between optical potential wells. By precisely controlling the optical forces and fluid drag forces in the microchannel, particles were made to hop between multiple optical spots, achieving the selection of bacteria and their specific antibodies. However, the loose overdamped system can cause micrometer-scale vibrations of particles at the capture site, leading to positional uncertainty. To address this issue, a team from Clarkson University in the USA [43] designed an adjustable phase gradient field to alter the optical forces. Under the combined action of optical forces and fluidic forces, particles of varying dimensions were captured at distinct locations and equilibrated via phase manipulation. In addition, the team from Nanyang Technological University in Singapore [44] also proposed a dual-waveguide near-field optical tweezers array configuration, capturing and selectively separating a large number of 100~500 nm particles by controlling the depth of the optical potential wells where different sized particles reside through the input laser power. Wang et al. [8] introduced a compact single-fiber optical tweezer-micropipette system. This system can categorize particles by shape and refractive index non-invasively, preserving the adaptability, high specificity, and accuracy inherent to optical fiber tweezers. Nie et al. [45] presented a comprehensive examination of the characteristics of bowtie-nanohole tweezers in the context of trapping and sorting nanoparticles, supported by theoretical analysis and numerical simulations.

However, existing optical tweezer systems tend to be complex in structure and costly, and they do not achieve continuous separation of cells. Starting from the theory of fiber optic waveguides, we utilized the T-matrix model to derive the scattering force calculation formula for single-mode fibers with flat end-faces. Based on this, we designed and fabricated an optofluidic chip with an expanded cross-channel structure and developed a single-mode fiber optic tweezers cell sorting system. The system successfully achieved continuous separation of yeast cells and polystyrene microspheres. In addition, we analyzed through simulations and experiments the effects of cell diameter, optical tweezer laser power, and fiber core diameter on cell displacement distance. These research findings provide new scientific evidence and technical methods for non-contact cell and particle sorting technologies.

## 2. Models

Based on the size relationship between the radius of microparticles and the wavelength of the laser, the accurate computation of optical trapping forces can be categorized into geometrical optics models, Rayleigh scattering models, and electromagnetic scattering models. However, as the radius of most cells is very close to the wavelength of the laser, the electromagnetic scattering model is mainly applicable. At present, the T-matrix approach is among the most efficacious and extensively applied methods for precisely calculating the scattering fields of individual or combined particles [46]. In the T-matrix method, once the calculation is performed, the results can be reused to calculate the radiation pressure of light without being affected by the particle’s position, orientation, or the characteristics of the incident light. Consequently, this study adopted the T-matrix algorithm.

Optical tweezers manipulate microparticles by transferring momentum via light, and the core mechanism involves the problem of light scattering. Both the incident light field and the scattered field satisfy the Helmholtz equation, and hence both fields are represented as a set of discrete basis functions [47], that is, the incident field is as follows:

(1) $$U_{inc}=\sum_{n}^{\infty }a_{n}\psi_{n}^{\left(inc\right)}$$

where *a_n_* is the expansion coefficients of the incident wave, and $\psi_{n}^{\left(inc\right)}$ are the basis functions.

The scattered field is as follows:

(2) $$U_{sca}=\sum_{k}^{\infty }p_{k}\psi_{k}^{\left(sca\right)}$$

where *p_k_* is the expansion coefficients of the scattered wave, and $\psi_{k}^{\left(sca\right)}$ are the basis functions.

As the scattered light and incident light have a linear relationship, the coefficients in Equations (1) and (2) can be expressed as a matrix equation,

(3) $$p_{k}=\sum_{n}^{\infty }T_{kn}a_{n}$$

where *T_kn_* is the T-matrix.

(4) $$T=-RgQ{\left[Q\right]}^{-1}$$

For specific calculation methods regarding **Q** and *Rg***Q**, please refer to reference [47]. If the T-matrix is known, the scattering fields with different expansion coefficients can then be calculated.

In a 3D optical potential trap, after interacting with light, particles experience two types of forces: the gradient force perpendicular to the optical axis and the scattering force along the direction of the optical axis. When the gradient force is greater than the scattering force, the particle is confined near the center of the focused light spot. When the scattering force exceeds the gradient force, the particle moves along the direction of the optical axis and eventually escapes from the light trap [48].

This paper mainly utilizes single-mode fiber-coupled laser to drive cells. The beam emerging from a flat-ended single-mode fiber has low convergence, which is characterized by weak focusing and primarily manifests as scattering force along the optical axis, thus making it suitable for cell propulsion.

When Gaussian laser light is coupled into a flat-ended single-mode fiber, the emerging light can be approximated to a Gaussian beam, with its waist located at the fiber’s end face. Assuming that the power transmitted through the fiber is P, the output intensity distribution of the flat-ended single-mode fiber is that of a Gaussian beam wavefront, with its waist situated on the surface of the fiber core, and the waist radius *ω_0_* is close to the size of the fiber core radius.

If the cell radius is r, and the distance from the center of the small sphere to the center of the fiber end face is r1=(x,y,z), then the electric field vector of the Gaussian beam is

(5) $$E\left(r1\right)=\hat{x}E_{0}\frac{ik\omega_{0}^{2}}{ik\omega_{0}^{2}+2z}\exp(-ikz)\times \exp\left[-i\frac{2kz(x^{2}+y^{2})}{{\left(k\omega_{0}^{2}\right)}^{2}+{\left(2z\right)}^{2}}\right]\times \exp\left[-\frac{\left(k\omega_{0}^{2})(x^{2}+y^{2}\right)}{{\left(k\omega_{0}^{2}\right)}^{2}+{\left(2z\right)}^{2}}\right]$$

where $\hat{x}$ represents the unit vector in the direction of beam polarization, *k* is the wavenumber of the surrounding medium, and *E*_0_ is the electric field strength at the waist center.

The field vectors of an electromagnetic field are real functions of time and space, and the transient energy flow through per unit area in unit time is represented by the Poynting vector as

(6) S(r1,t)=E(r1,t)×H(r1,t)=12ReE(r1)×H(r1)exp(iωt)+12ReE(r1)×H(r1)

Assuming the center of the microsphere is in the same plane as the fiber core, by solving it using the T-matrix model, the formula for the axial scattering force experienced by the cell can be obtained as

(7) $$F_{s}=\frac{2r^{2}\pi^{2}\omega_{0}^{2}PQ_{pr}}{c(\pi^{2}\omega_{0}^{4}+z^{2}\lambda^{2})}$$

where *Q_pr_* is the radiation pressure coefficient [49], *c* is the speed of light, $\omega_{0}$ is the beam waist radius, *P* is the laser power, and *z* is the distance of the cell from the fiber end face.

From the formula, it can be seen that the scattering force experienced by the cell primarily depends on the laser power, cell radius, radiation pressure coefficient, cell position, beam waist radius, and wavelength. When a single-mode fiber is used to couple laser light to drive cells, if the power, wavelength, cell position, and beam waist radius are fixed, the scattering force mainly depends on the cell radius. Under the same conditions, the larger the cell radius, the greater the scattering force it experiences; conversely, the smaller the cell radius, the lesser the scattering force. For cells of the same size, the greater the applied laser power, the greater the force experienced.

## 3. Materials and Methods Results

### 3.1. Experimental Setup

**Figure 1:**
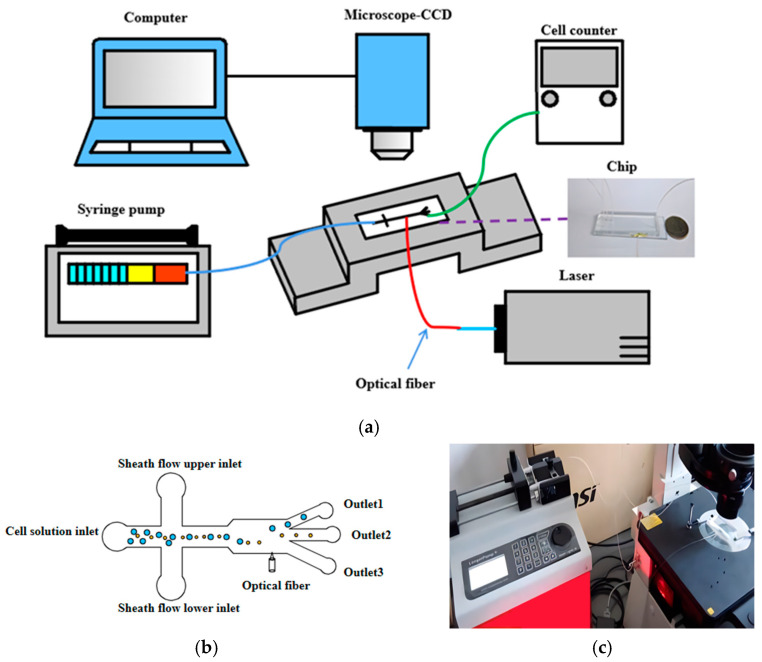
(**a**) Cell experiment sorting platform, (**b**) principle of Sorting, and (**c**) local view of the microfluidic system.

The cell sorting experimental platform primarily includes the following equipment: an optofluidic chip, a micro-injection pump, a microscope, a high-speed camera, a pump light source, a cell counter, and related accessories, as shown in Figure 1a. The basic principle is as follows: under the action of the micro-injection pump, the cell solution is introduced into the main channel, while the buffer solution enters the sheath flow channel. The sheath flow from both sides compresses the cell solution to form a single-cell stream. As cells of different sizes pass through the fiber-optic end face interaction area, the laser’s scattering force drives the cells to move laterally along the channel by varying distances, resulting in their exit through distinct outlets, as depicted in Figure 1b. The other end of the pigtail embedded in the optical fiber chip needs to be connected to the laser through a collimating lens. The optical fiber chip is horizontally fixed on the stage of the microscope. The microscope is equipped with a CCD high-speed camera and a computer for convenient data transmission, allowing for easier observation and recording of the cells’ movement state through the computer. Figure 1c illustrates the local view of the microfluidic system.

### 3.2. Fabrication of the Optofluidic Chip

**Figure 2:**
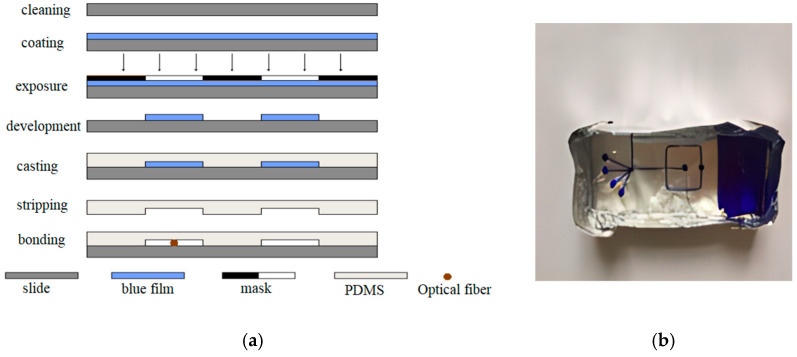
(**a**) Schematic diagram of the optofluidic chip fabrication steps and (**b**) PDMS curing.

The optofluidic chip is formed by bonding a PDMS cover slip to an ITO substrate. The PDMS cover slip is fabricated using soft lithography techniques to create micrometer-scale channels on the chip surface, which primarily involve the production of chip masks, fabrication of positive molds, casting and curing of PDMS, and vacuum plasma bonding, as shown in Figure 2.

#### 3.2.1. Channel Structure Design

The cross-sectional shape of the microchannel is fundamental to channel design and fabrication. Based on the chip fabrication process, the flow characteristics of the cell solution within the microchannel, and the requirements for optical actuation, the microchannel cross-section is designed to be rectangular. The geometric dimensions of the microchannel have a significant impact on fluid flow within the microfluidic chip. The width and depth of the channel should not be too large. The design features a typical cross-channel with a uniform width of 100 µm, an inlet channel length of 200 µm, and a separation channel length of 800 µm. In consideration of the need for optical tweezers to overcome Stokes’ drag force to manipulate cells and slow down their motion during sorting, expand the sorting channel to 200 µm. Considering that cells may settle at the bottom of the channel due to gravity or form layered flow during movement, which can complicate optical actuation, and since the chip needs to embed single-mode optical fibers with a cladding diameter of 125 µm, to reduce fabrication complexity and cost, the depth of the main channel, sheath flow channel, and fiber channel were all designed to be 120 µm.

Additionally, the pre-embedded position of the optical fiber, which is the location of the fiber channel, must be determined. According to the inlet section effect, if the fiber channel is very close to the intersection of the cross-channel, the liquid flow will be unstable, and the position of cell movement will be uncertain, preventing cells from being deprived of scattered light and resulting in unsatisfactory sorting. However, if the fiber position is too far from the nozzle, the focused flow may deform due to diffusion. Therefore, the position where the fiber is embedded should be in a stable part of the liquid flow not far from the nozzle. Based on the size of the chip and the position of the separation outlet, we set the fiber channel 500 µm away from the nozzle.

#### 3.2.2. Bonding and Pretreatment

**Figure 3:**
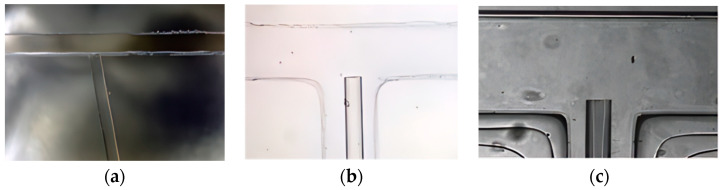
Optical fiber embedding effect diagram: (**a**) the “pre-embedding of the fiber” method, (**b**) the “threading” method, and (**c**) the “direct bonding” method.

In the fabrication process of the optofluidic chip, the most challenging aspect is how to embed the optical fiber. In experiments, our research group has successively used three methods to embed the fiber into the chip: the “pre-embedding of the fiber” method, the “threading” method, and the “direct bonding” method. The effects of these embedding methods are shown in Figure 3. Among them, “direct bonding” method yields the best results. This method requires the embedding of optical fibers during the chip bonding process. Therefore, during mask production, a fiber channel is reserved for easy positioning of the fiber. The specific steps are as follows: quickly place the PDMS cover slip taken out from the bonding machine under a microscope with the focus adjusted, then use a pipette to drop several drops of ethanol on the reserved fiber channel, observe it under the microscope, place the single-mode optical fiber in the appropriate position within the reserved fiber channel, quickly cover it with the plasma-activated glass slide and press down with external force, and seal the ports of the reserved fiber channel with epoxy resin. Finally, place the chip on a heating stage at 80 degrees Celsius for 2 min to form a permanent bond. As seen in Figure 3c, the embedding effect was good, and the fiber end face was clean.

After the chip is fabricated, pretreatment of the microchannel is required before the experiment. To prevent cells from adhering to the surface of the microfluidic channel, manually inject a small amount of surfactant (tween 20) into the channel using a syringe, then rinse the channel repeatedly three times with deionized water, and finally extract all the liquid from the channel [50].

## 4. Analysis and Discussion

### 4.1. FE Analysis

To study the effect of scattering forces on cells, COMSOL Multiphysics 6.0 was used to simulate the sorting effect. In COMSOL Multiphysics 6.0 software, laminar flow, electric current, particle tracing, and electromagnetic field were added to perform a coupled simulation analysis of the motion states of cells with diameters of 10 µm and 20 µm under different fiber laser conditions. A transient solver was used to fully couple the three physical fields of laminar flow, electric current, and electromagnetic waves. The optical scattering force, weakly coupled from the solution, was integrated into the fluid particle tracing module, and through post-processing, different deflection distances were obtained, thereby simulating the sorting of particles or cells. Specific parameter settings are shown in Table 1.

**Figure 4:**
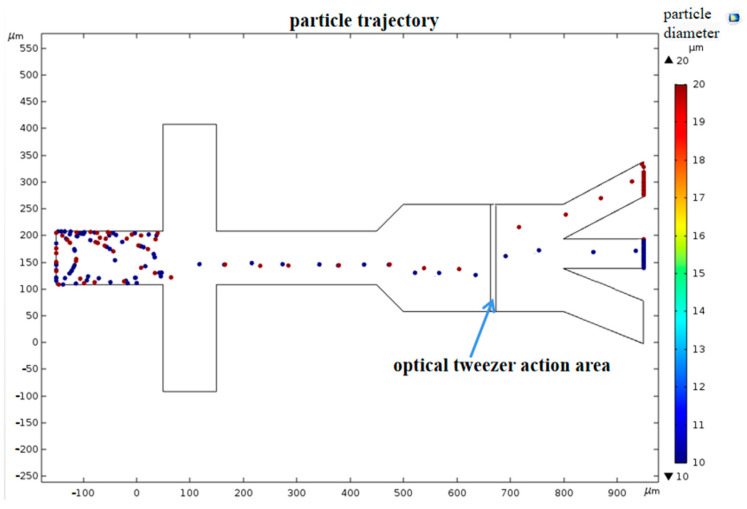
The trajectory diagram for sorting particles after they are subjected to scattering forces.

**Figure 5:**
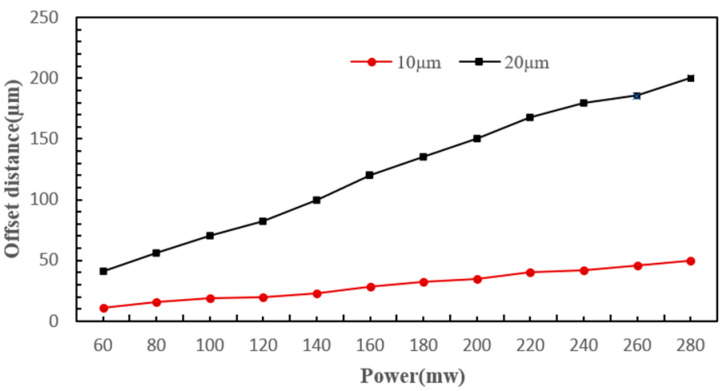
The influence of different powers on the deflection distance.

**Figure 6:**
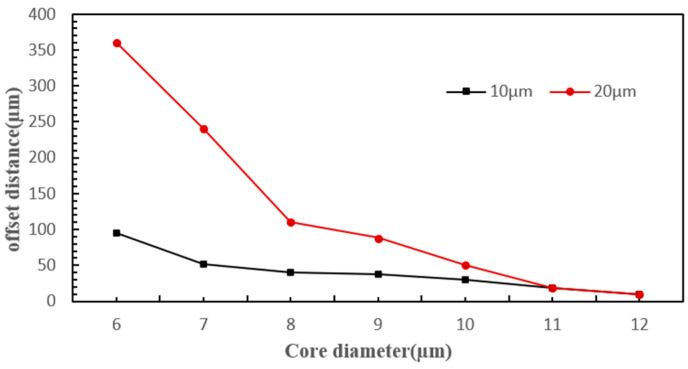
The effect of varying fiber core diameters on the deflection distance.

Figure 4 shows that when particles move into the fiber optic action area, they experience varying scattering forces from the same laser due to their different diameters. This results in distinct displacements of the two types of particles along the laser’s axis, leading them to enter different isolation channels. During the coupling of the four physical fields, a parametric sweep method was used to solve for the deflection distances of particles with diameters of 10 µm and 20 µm at laser powers ranging from 0 to 300 mW, and fiber core diameters ranging from 6 µm to 12 µm. Figure 5 and Figure 6 illustrate the impact of varying powers and fiber core diameters on offset distance, respectively.

From the figures, it can be seen that under the same conditions, particles of different diameters moved different distances along the optical axis after being subjected to optical scattering forces; the larger the diameter, the greater the distance of movement. As the laser power increased, the particle offset distance also increased, showing a linear relationship. However, excessively high laser power caused a large difference in the offset distances of the two types of cells and may damage the cells, while too low laser power resulted in too small a difference in cell deflection distances, which is not conducive to sorting. As the fiber core diameter increased, the particle offset distance became smaller, and the decrease in offset distance for the 20 µm diameter particles was more significant, indicating that the scattering force experienced by the particles was inversely proportional to the fiber core diameter. When the fiber core diameter was small, the difference in offset distance between the two types of cells was larger, but when the fiber core diameter was large (such as 10 µm), the difference in offset distance was too small, and even when the fiber core diameter was 12 µm, the offset distances of the two types of particles were essentially the same. Therefore, larger fiber core diameters were not conducive to cell sorting. Taking into account the aforementioned factors, subsequent experiments were conducted using a single-mode fiber with an 8 µm core diameter that delivered a 180 mW laser for cell sorting.

### 4.2. Separation Efficiency

To reflect the motion states of cells with different diameters under scattering forces, experiments were conducted using a mixed solution containing yeast cells (diameter size of 8~10 µm) and polystyrene microspheres (diameter size of 15~20 µm). Before injecting the reagent into the chip, it was subjected to an ultrasonic agitator for 5 min to ensure the uniform distribution of particles within the reagent. The reagent was injected into the chip through a plastic hose. Particle tracking was facilitated by an optical microscope in conjunction with a high-speed CCD camera, while a cell counter assessed the variety, quantity, and viability of particles within the gathered solution at various outlets.

The key to driving cells with laser through an optical fiber lies in achieving high coupling efficiency between the laser and the fiber. This is primarily accomplished by focusing a laser beam using an objective lens with a high numerical aperture and then adjusting the platform to couple the focused laser into the fiber core, thus driving the cells in suspension. Near-infrared lasers with wavelengths ranging from 700 nm to 1400 nm are widely used in biomedical and other fields. Therefore, after repeated cell driving experiments within this wavelength range, it was found that the 980 nm single-mode pump source (wavelength 980 nm, output power 0~300 mV) can perfectly achieve the coupling of the output laser with the fiber by connecting one end of the pigtail to the laser and embedding the other bare fiber end into a microfluidic chip. Consequently, a laser wavelength of 980 nm was chosen.

**Figure 7:**
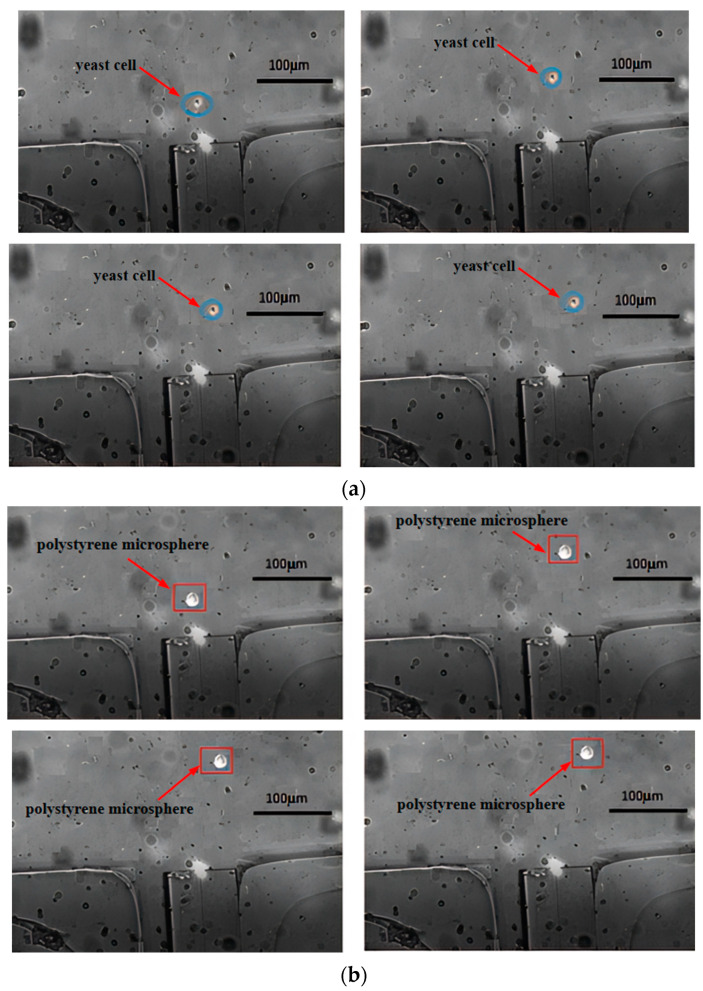
Motion trajectory under 180 mW optical tweezers: (**a**) yeast cells and (**b**) polystyrene microspheres.

The main channel was perfused with a mixed cell solution, while the sheath flow channels were perfused with a 0.9% mass fraction NaCl solution. The micro-injection pump was adjusted to set inlet velocities. The laser was turned on, outputting power at 180 mW with a wavelength of 980 nm. Figure 7 shows the motion trajectory plots of yeast cells and polystyrene microspheres, after being subjected to optical tweezers scattering forces. From both figures, it can be observed that when passing through the fiber end face, the particles moved upwards to the right under the combined effects of Stokes force and light scattering force (other forces acting on the particles are negligible). However, during the first second, the scattering force dominated, resulting in a significant longitudinal displacement along the optical axis. In the following two seconds, as the particles gradually left the range of the optical trap, the Stokes force became dominant, leading to a noticeable transverse motion. Comparing Figure 7a,b, it is evident that polystyrene microspheres travelled a greater distance along the optical axis than yeast cells and at a higher speed due to their larger diameter and consequently greater scattering force. Therefore, after escaping from the range of the optical trap, both types of particles can enter different separation channels under the influence of Stokes force. The sorting efficiency measured by the cell counter can reach 86%, and about 90% of the yeast cells sorted still have cell activity.

### 4.3. Parameter Analysis

#### 4.3.1. Cell Diameter

Figure 7 illustrates that upon exposure to the scattering force of optical tweezers, various cells moved different distances along the axial direction. The polystyrene microspheres moved approximately 120 µm in the axial direction, whereas the yeast cells moved about 30 µm, which means the movement distance of the polystyrene microspheres is about four times that of the yeast cells. Under the same conditions, larger diameter cells experienced greater scattering force and consequently moved a greater distance along the axis, whereas smaller diameter cells, experiencing less scattering force, moved a shorter distance along the axis. According to the analysis based on the scattering force formula, this is because the scattering force experienced by a particle is proportional to the square of the particle’s radius.

#### 4.3.2. Laser Power

**Figure 8:**
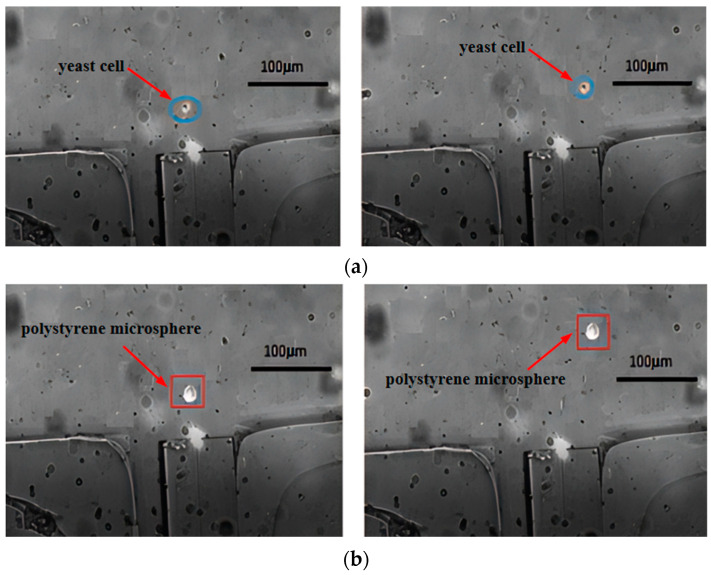
The offset distance under 120 mW optical tweezers: (**a**) yeast cells and (**b**) polystyrenemicrospheres.

**Figure 9:**
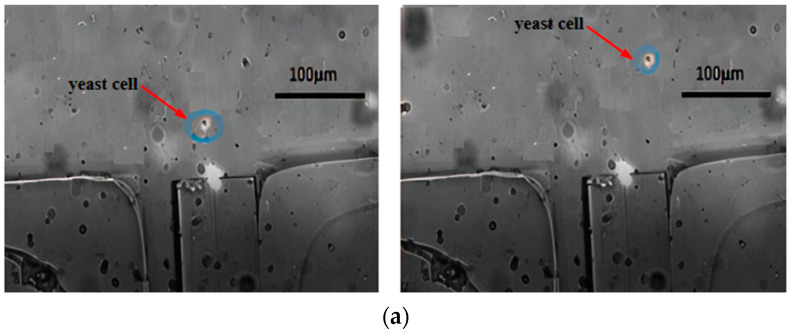

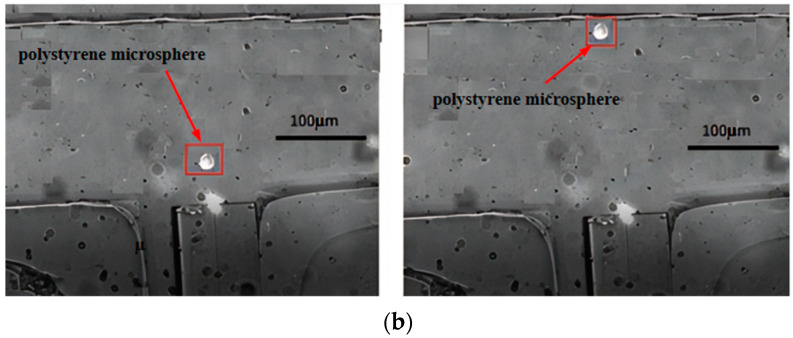
The offset distance under the action of 240 mW optical tweezers: (**a**) yeast cells and (**b**) polystyrene microspheres.

Based on the simulation results, experiments were conducted in this paper on the effects of optical fiber tweezers at powers of 120 mW, 180 mW, and 240 mW on different cells. Figure 8 and Figure 9 show the deflection distances of yeast cells and polystyrene microspheres under the influence of optical fiber tweezers at powers of 120 mW and 240 mW, respectively. Under the influence of an optical tweezer at 120 mW, yeast cells and polystyrene microspheres exhibited shifts of approximately 20 µm and 70 µm, respectively. The small difference in displacement distance between the two particles is not conducive to sorting. However, at 240 mW, yeast cells and polystyrene microspheres shifted by about 40 µm and 180 µm, respectively. The large difference in displacement distance is also unfavorable for sorting, as the polystyrene microspheres were nearing the channel walls. By comparing Figure 7, it was found that the greater the power, the greater the deflection distance for the same type of cell, showing a roughly linear increase. Moreover, when subjected to a laser of the same power, polystyrene microspheres exhibited greater lateral displacement than yeast cells. This was because the scattering force experienced by a particle is directly proportional to the laser power.

#### 4.3.3. Fiber Core Diameter

**Figure 10:**
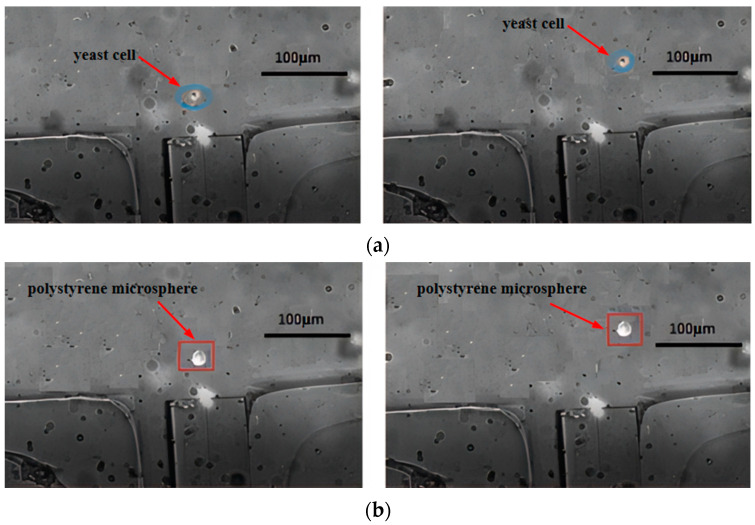
The offset distance under optical tweezers with a core diameter of 10 µm: (**a**) yeast cells and (**b**) polystyrene microspheres.

Next, experiments were conducted on the effect of optical fiber tweezers with fiber core diameters of 8 µm and 10 µm on different cells. Figure 10 shows the deflection results of yeast cells and polystyrene microspheres under the influence of optical fiber tweezers with a 10 µm fiber core diameter. From the figures, it can be seen that the yeast cells moved approximately 20 µm under the action of the optical tweezers, while the polystyrene microspheres moved about 40 µm. The small difference in displacement distance between the two particles is not conducive to sorting. Comparing Figure 7, it was found that the larger the fiber core diameter, the smaller the deflection distance, and the deflection distance of the polystyrene microspheres decreased more significantly with the increase in fiber core diameter. This indicates that the scattering force experienced by the cells was inversely proportional to the fiber core diameter, which is consistent with the scattering force formula.

## 5. Conclusions

Our group investigated the mechanism of cell sorting using flat-end optical tweezers, proposing a method for cell sorting that is easily integrable and maintains high cell viability.

The main conclusions are as follows:

(1) The scattering force experienced by cells primarily depends on laser power, cell radius, radiation pressure coefficient, cell position, beam waist radius, and wavelength. Hence, cells of different diameters experience varying scattering forces from the same laser, causing them to shift different distances along the laser’s axis and thus enter distinct sorting channels.
(2) After repeated experiments, we successfully achieved the effective integration of single-mode fibers with microfluidic chips, fabricated an optofluidic chip, and established a cell sorting experimental platform. We optimized the traditional cross-channel chip structure, creating an expanded cross-channel that enabled continuous sorting of yeast cells and polystyrene microspheres. Compared to other sorting systems, this system does not require labeling and can achieve high-viability cell sorting.
(3) Through simulations and experiments, we analyzed the effects of various parameters such as cell diameter, optical tweezers laser power, and fiber core diameter on the cell displacement distance, validating the rationality of the formula.

## Figures and Tables

**Table 1:** Main parameters.

Parameter	Value
Particle diameter 1 d_1_/µm	10
Particle diameter 2 d_2_/µm	20
Laser power/mW	180
Laser wavelength/[nm]	980
Fiber core diameter d_f_/µm	8
Main channel inlet velocity/µm/min	1
Sheath flow upper inlet velocity/µm/min	1.2
Sheath flow lower inlet velocity/µm/min	1

## Data Availability

The data presented in this study are available on request from the corresponding author.
